# Supplementary material for: Persistence and reversal of plasmid-mediated antibiotic resistance
Source: Nat Commun. 2017 Nov 22;8:1689. doi: 10.1038/s41467-017-01532-1 (PMC5698434; doi:10.1038/s41467-017-01532-1)
Supplement: Supplementary file 1 — Supplementary Information [file 41467_2017_1532_MOESM1_ESM.pdf]

Supplementary Methods

Nomenclature

To facilitate reading, naming convention of species (S) and plasmid (P) combinations are kept consistent throughout. Namely, S^P denotes a background species S carrying plasmid P, S^0 indicates S in the absence of P, and S without a superscript indicates the generic species either with or without the plasmid. In the case where the plasmid P is non-transferrable by conjugation, S^{P-} is used, where "-" indicates the lack of transfer capabilities.

Plasmid Loss Calculations

Experimentally, we measure plasmid loss by propagating a population initiated with 100% plasmid-carrying cells that are non-transferrable, and quantifying the percentage of plasmid-carrying cells over time. The decay of the fraction of plasmid carrying cells (B^1) is measured using the following equation:

$$\frac{B^1}{B^1+B^0} = x_1 e^{-x_2 t} \ ,$$

where the rate constant x_2 represents the *observed* plasmid loss rate (κ_{obs}). Since the plasmid is non-transferrable, conjugation does not confound these measurements. However, since the plasmid is costly, κ_{obs} is a function of the cost as well, since the effects of competition cannot be excluded.

To determine the extent to which cost confounds the plasmid loss rate constant, consistent with our experimentally setup we assume:

1. Cells grow approximately exponentially
2. $\eta_C = 0$

The differential equations can be written as follows:

$$\frac{dB^1}{dt} = \mu_1 B^1 - B^1\kappa - DB^1$$
$$\frac{dB^0}{dt} = \alpha\mu_1 B^0 + B^1\kappa - DB^0 \ .$$

This system of differential equations can be solved analytically, such that:

$B^1 = e^{\lambda t}$, where $\lambda = \mu_1 - \kappa - D$, and

$$B^0 e^{t(D-\alpha\mu_1)} = \int \kappa e^{t(\lambda+D-\alpha\mu_1)} dt$$
$$B^0 = \frac{\kappa}{D-\alpha\mu_1+\lambda} \ e^{t(\lambda+D-\alpha\mu_1)} + C_1 e^{t(\alpha\mu_1-D)}$$
$$C_1 = -\frac{\kappa}{D-\alpha\mu_1+\lambda}$$
$$B^0 = \frac{\kappa}{D-\alpha\mu_1+\lambda}\left(e^{t\lambda} - e^{t(\alpha\mu_1-D)}\right)$$

From the solution of B^0, we can see that if the plasmid has a low cost (e.g. $\alpha \approx 1$, which is true for plasmid K (α =1.02) then

$$B^0 \approx \left(e^{t(\alpha\mu_1-D)} - e^{t\lambda}\right)$$

Therefore, substituting B^1 and B^0 with the explicit form, we have:

$$\ln(B^1) - \ln(B^1 + B^0) = \ln(x_1) - x_2 t$$

$$t(\lambda + x_2) = \ln(x_1) + \ln\left(e^{t\lambda} - e^{t\lambda} + e^{t(\alpha\mu_1 - D)}\right)$$

This gives us the final equation, where $x_2 = \kappa_{obs}$:

$$\kappa_{obs} = \kappa + \mu_1(\alpha - 1) + \frac{\ln(x_1)}{t}$$

This shows that κ_{obs} is most accurate as $\alpha \rightarrow 1$ (small fitness effect), e.g. $\kappa_{obs} \approx \kappa$. As the cost increases, κ_{obs} is confounded by the growth rate. In that case, $\kappa = \kappa_{obs} + \Delta_p$, where $\Delta_p = \mu_1(\alpha - 1) + \frac{\ln(x_1)}{t}$. According to our estimates, for x_1=0.96 (flow detection limit) and small time (e.g. t=10 for relevant range of α) then $\Delta_p \approx 0.0019$, which is less than the error associated with estimating κ_{obs} (≈ 0.0022).

Model Development

A. One-species, one-plasmid model

The general kinetic model for conjugation dynamics consists of $n2^p$ ordinary differential equations (ODE), where n is the number of species (S) and p is the number of transferrable plasmids. For the simplest case, two ODE equations are used to represent 1 species divided into 2 sub-populations (n = 1, p =1) that either does (s^1) or does not (s^0) carry the plasmid:

$$\frac{ds^0}{dt} = \alpha\mu s^0\left(1 - \frac{s^0 + s^1}{N_m}\right) - \eta s^0 s^1 + \kappa s^1 - D s^0, \qquad \text{Eq 1}$$

$$\frac{ds^1}{dt} = \mu s^1\left(1 - \frac{s^0 + s^1}{N_m}\right) + \eta s^0 s^1 - \kappa s^1 - D s^1 . \qquad \text{Eq 2}$$

In this model, s^0 can gain the plasmid through conjugation at the conjugation efficiency rate η, and s^1 can lose the plasmid due to natural segregation error at a rate constant κ. The two populations grow to a shared carrying capacity (N_m) and the growth rate of s^0 is proportional to the growth rate of s^1 (μ) according to α such that s^0 grows at a rate $\alpha\mu$. Thus, α represents the relative burden of the plasmid when $\alpha > 1$, and the relative benefit when $\alpha < 1$. D represents the continuous dilution rate of both populations. Experimental measurements for all parameters can be found in Supplementary Table 2. Letting $S^1 = \frac{s^1}{N_m}$, $S^0 = \frac{s^0}{N_m}$ and $\eta_c = N_m\eta$, we get:

$$\frac{dS^0}{dt} = \alpha\mu S^0(1 - S^0 - S^1) - \eta_C S^1 S^0 + \kappa S^1 - D S^0 , \qquad \text{Eq 3}$$

$$\frac{dS^1}{dt} = \mu S^1(1 - S^0 - S^1) + \eta_C S^1 S^0 - \kappa S^1 - D S^1 . \qquad \text{Eq 4}$$

To ensure that a continuous dilution rate is an appropriate approximation of the discrete 24-hour dilutions that were performed experimentally, we implemented a variant of

this model without dilution, i.e. Supplementary Eq 3 and 4 but with D=0. Here, every 24 hours the final fraction of S^1 and S^0 is collected, divided by 10,000, and used to initiate the next seasonal growth period. Results show qualitatively similar trends regardless of whether continuous or periodic dilution is used (Supplementary Fig. 1D), although notably the time-scale changes slightly due to the different average growth rate experienced throughout the duration of the entire time course. Since the continuous model approximates a continuous growth rate below the carrying capacity, we use a higher growth rate in the discreet model (1.2 hr^{-1}), but use consistent values for the comparison of the continuous model (e.g. all columns in Supplementary Fig. 1D use μ=1.2 hr^{-1}), demonstrating this is not dependent on the dilution method. All other parameters are unchanged. Given the consistency, Supplementary Eq 3 and 4 with continuous dilution are used to simplify analysis. Importantly, the continuous dilution format reduces the model sensitivity to growth rate as long as it is sufficiently greater than D.

To examine the potential influence of compensatory adaptation, we modified this model to include a third population of mutants, M:

$$\frac{dS^0}{dt} = \alpha\mu S^0(1 - S^0 - S^1 - M) - \eta_C S^1 S^0 + \kappa(M + S^1) - DS^0,$$
$$\frac{dS^1}{dt} = \mu S^1(1 - S^0 - S^1 - M) + \eta_C S^1 S^0 - \kappa S^1 - DS^1 - \beta S^1 ,$$
$$\frac{dM}{dt} = \alpha_M \mu S^1(1 - S^0 - S^1 - M) + \eta_C M S^0 - \kappa M - DM + \beta S^1 . \qquad \text{Eq 5}$$

S^1 transitions to M at a rate β, such that M has reduced fitness burden and grows at a rate equal to (α_M=1) or slightly greater than (α_M=0.9) S^0. Analysis demonstrates that regardless of whether the plasmid can conjugate, a rapid transition rate ($\beta \approx 0.001$ and above) will result in a significant portion of the final plasmid population consisting of M compared to S^1 after 14 days (Supplementary Fig. 2B). This value is large compared to typical estimates of mutation rates. Particularly, it is estimated that spontaneous mutations arise at a frequency of around 10^{-9} per base pair per generation[1], and only a fraction of these will confer increased resistance. In our study, the plasmid was readily eliminated in the absence of conjugation (Fig. 2B). Thus, this analysis suggests that compensatory mutations of this low-cost plasmid likely did not contribute significantly to the overall dynamics.

B. Deriving a simple stability criterion

Here we consider the situation where the plasmid is costly (α > 1). When $\eta_C > 0$, in the limiting case of $\frac{\kappa}{S^0}$ being small, we can solve Eq 3-4 for the steady state of S^0:

$$S^0 = \frac{(\alpha\mu^2 - \mu D) - (\mu - D - \kappa)(\alpha\mu + \eta_C)}{\alpha\mu^2 - (\alpha\mu + \eta_C)(\mu - \eta_C)}$$

From this solution, we note that S^0 decreases with η_C. When η_C is above a threshold (η_{Crit}), S^0 drops below 0. This point corresponds to an approximate and conservative estimate of the conjugation efficiency to allow persistence of the plasmid (i.e. the plasmid exists and S^1 dominates). We determine this critical conjugation efficiency η_{Crit}, by setting S^0 = 0:

$$(\alpha\mu^2 - \mu D) - (\mu - D - \kappa)(\alpha\mu + \eta_{Crit}) = 0$$
$$\eta_{Crit}(\mu - D - \kappa) = (\alpha\mu^2 - \mu D) - \alpha\mu(\mu - D - \kappa)$$
$$\eta_{Crit}(\mu - D - \kappa) = \mu(\alpha(D + \kappa) - D)$$
$$\eta_{Crit} = \frac{\mu(\alpha(\kappa+D)-D)}{\mu-D-\kappa}$$

Assuming $\mu \gg \kappa + D$, and we get:

$$\eta_C > \eta_{Crit} = \alpha(\kappa + D) - D \qquad \text{Eq 6}$$

Eq 6 is a stronger criterion than the minimum conditions necessary for plasmid existence, as described by Stewart and Levin (see main text, e.g. S^1>0), where $\eta_{Crit} = (\kappa + D) - \frac{D}{\alpha}$. Indeed, the latter criterion defines a lower bound on the efficiency required for plasmid existence. In comparison, our criterion provides a conservative upper estimate to facilitate both experimental interpretation and cost-dependent parameter estimation.

All modeling parameters can be found in Supplementary Table 2. To mimic experimental conditions, each simulation set consists of a set of 20 replicates that vary in their initial fraction. In MATLAB, this is achieved by initiating each simulation with generating a vector of randomly assigned initial cell densities for each population drawn from a uniform distribution. This is done such that the total density is maintained at $1\text{x}10^{-6}$. The same vector of randomly generated initial conditions is maintained for a single simulation set, i.e. for comparing different α. To account for error estimates and day-to-day variability in conjugation efficiencies measurements, we introduce noise such that all efficiencies for each set of initial conditions can vary a small amount from the basal value, within 10% of the mean. This variability does not change the qualitative results (Supplementary Fig. 2C). All parameter values are based on experimental estimates (Supplementary Table 2). This model was used for Fig. 2B and Fig. 3B. To model the plasmid with increased cost (C, Fig. 3B), the only parameters that changed were α (experimentally estimated) and κ (assumed a two-fold increase based on cost increase).

C. Modeling populations with multiple species and/or plasmids

I. Two-species, one-plasmid model

Modeling two species with one plasmid (n = 2, p = 1) required four ODEs, where the subscript 1 or 2 indicates species (S) number and α_i represents the respective cost of the plasmid in each respective species (quantified experimentally in Supplementary Fig. 4A):

$$\frac{dS_1^0}{dt} = \alpha_1 S_1^0 \mu_1 \left(1 - \frac{S_1^0+S_1^1+S_2^0+S_2^1}{N_m}\right) - \eta_1 S_1^0 S_1^1 - \eta_2 S_1^0 S_2^1 + \kappa S_1^1 - D S_1^0 \qquad \text{Eq 7}$$
$$\frac{dS_1^1}{dt} = S_1^1 \mu_1 \left(1 - \frac{S_1^0+S_1^1+S_2^0+S_2^1}{N_m}\right) + \eta_1 S_1^0 S_1^1 + \eta_2 S_1^0 S_2^1 - \kappa S_1^1 - D S_1^1 \qquad \text{Eq 8}$$
$$\frac{dS_2^0}{dt} = \alpha_2 S_2^0 \mu_2 \left(1 - \frac{S_1^0+S_1^1+S_2^0+S_2^1}{N_m}\right) - \eta_3 S_2^0 S_2^1 - \eta_4 S_2^0 S_1^1 + \kappa S_2^1 - D S_2^0 \qquad \text{Eq 9}$$
$$\frac{dS_2^1}{dt} = S_2^1 \mu_2 \left(1 - \frac{S_1^0+S_1^1+S_2^0+S_2^1}{N_m}\right) + \eta_3 S_2^0 S_2^1 + \eta_4 S_2^0 S_1^1 - \kappa S_2^1 - D S_2^1 \qquad \text{Eq 10}$$

Each simulation is initiated by generating a vector of randomly assigned initial cell densities for each population drawn from a uniform distribution such that the total density for all four populations is maintained at $1x10^{-6}$.

II. One-species, two-plasmid model

To model one species with two plasmids (*n*=1, *p*=2), we used indexed superscripts to indicate which plasmid is being transferred and received. Thus, superscript 00 indicates no plasmid, superscripts 01 and 10 indicate the species carries either one of the plasmids, and 11 indicates the species carries both plasmids. The cost for each plasmid individually was determined previously, where α_1 and α_2 are the same as values in the previous model. The cost for the species carrying both plasmids was quantified similarly as with all other cost experiments (Supplementary Fig. 4B).

Let $p = \frac{S^{00}+S^{01}+S^{10}+S^{11}}{N_m}$

$$\frac{dS^{00}}{dt} = S^{00}\mu(1-p) - S^{00}(\eta_1 S^{01} + \eta_2 S^{10} + 2\eta_3 S^{11}) + \kappa_{01}S^{01} + \kappa_{10}S^{10} - DS^{00} \quad \text{Eq 11}$$

$$\frac{dS^{01}}{dt} = \frac{S^{01}\mu}{\alpha_K}(1-p) + \eta_3 S^{00}S^{11} + \eta_1 S^{01}S^{00} - \eta_4 S^{10}S^{01} - \eta_5 S^{11}S^{01} + \kappa_{10}(S^{11} - S^{01}) - DS^{01} \quad \text{Eq 12}$$

$$\frac{dS^{10}}{dt} = \frac{S^{10}\mu}{\alpha_C}(1-p) + \eta_3 S^{00}S^{11} + \eta_2 S^{00}S^{10} - \eta_4 S^{10}S^{01} - \eta_6 S^{11}S^{10} + \kappa_{10}(S^{11} - S^{10}) - DS^{10} \quad \text{Eq 13}$$

$$\frac{dS^{11}}{dt} = \frac{S^{11}\mu}{\alpha_{CK}}(1-p) + 2\eta_4 S^{10}S^{01} + \eta_5 S^{11}S^{01} + \eta_6 S^{11}S^{10} - \kappa_{10}S^{11} - \kappa_{01}S^{11} - DS^{11} \quad \text{Eq 14}$$

Each simulation is initiated by generating a vector of randomly assigned initial cell densities for S^{01} and S^{10} only, drawn from a uniform distribution such that the total density is maintained at $1x10^{-6}$.

III. Three-species, three-plasmids model

To model the three-species three-plasmid community (*n*=3, *p*=3), we use superscript indices to represent the plasmid, where the index represents which plasmid the species carries, and subscripts indicate the species number (e.g. S_2^{011} is species 2 carrying plasmids 2 and 3). This notation allowed us to create an algorithm for automatically generating the ordinary differential equations for any arbitrary number of species or plasmids.

For this case, $n2^P$ = 24 sub-populations that result from three species (S_1 S_2, S_3) carrying up to three plasmids, where each plasmid is initiated in a unique species (S_1^{100}, S_2^{010}, S_3^{001}), and these *6* populations only makeup the starting composition. Each species and plasmid pair has a unique α associated with it, estimated from Supplementary Fig. 5B. All plasmid loss rates are assumed to be equal such that $\kappa = 0.001$ hr^{-1}, and dilution is the same as all previous simulations (0.05 hr^{-1}).

A sample equation (Supplementary Eq 15) is shown below for S_1^{000}, and a generalized form of the equation is presented in Supplementary Eq 16 (see section 'Generalized conjugation model' below and Supplementary Table 4 for detailed description of equation terms and derivation):

$$\frac{dS_1^{000}}{dt} = \mu S_1^{000}\left(1 - \sum_{i=1}^{3}\sum_{a=0}^{1}\sum_{b=0}^{1}\sum_{c=0}^{1} S_i^{abc}\right) \quad \text{(logistic growth)}$$

$$-\eta S_1^{000}(S_1^{001} + S_1^{010} + 2S_1^{011} + S_1^{100} + 2S_1^{101} + 2S_1^{110} + 3S_1^{111} + S_2^{001} + S_2^{010} + 2S_2^{011} + S_2^{100} + 2S_2^{101} + 2S_2^{110} + 3S_2^{111} + S_3^{001} + S_3^{010} + 2S_3^{011} + S_3^{100} + 2S_3^{101} + 2S_3^{110} + 3S_3^{111}) \quad \text{(loss by conjugation)}$$

$$+\kappa(S_1^{001} + S_1^{010} + S_1^{110}) \quad \text{(gain by plasmid loss from other populations)}$$

$$-DS_1^{000} \quad \text{(dilution)} \qquad \text{Eq 15}$$

η is assumed to be constant for generality purposes. To assign all 252 parameters of conjugation efficiency in the simulations, we randomly generate values that fall within 10% of the basal value 0.025 hr^{-1}. To estimate α, we measured the cost for all three populations, each carrying a single plasmid. Supplementary Table 2 lists each of these values individually. To implement the model, we assume for generality that α for carrying more than one plasmid is equal to the minimum cost value associated with each plasmid being carried, determined by our experimental estimations. We note that unlike the simplest model (one-species, one-plasmid), this scenario has various alternative steady states determined largely by the combination of conjugation efficiencies and plasmid costs. Therefore, it is possible that randomly chosen initial conditions may result in plasmid loss through competition.

Generalized conjugation model

To derive a general formula, we let N be the number of species, p be the number of plasmids, such that there are $N2^p$ total populations (all species/plasmid combinations). Note that conjugation can be algorithmically implemented by matrix operations between vectors of unique sets of plasmids. To facilitate writing the equation, we introduce a set of definitions and well-defined linear algebra operations.

Let $z = 2^p - 1$ be the number of unique plasmid combinations indexed by $0 \le j \le z = 2^p - 1$. Then we define $\gamma_j = [b_1 \quad b_2 \quad \cdots \quad b_p], \forall j$ be the row vector consisting of binary elements such that $b_i = \begin{cases} 1 & plasmid \\ 0 & no\ plasmid \end{cases}$. Therefore, the set of all γ_j ($\{\gamma_j\}$) consists of the ordered set of all possible unique plasmid combinations represented as binary vectors. For example, γ_0 is the first vector in $\{\gamma_j\}$ that consists of all zeros (no plasmids), and γ_z is the last vector in $\{\gamma_j\}$ and represents a vector of all ones. Thus, $S_n^{\gamma_0}$represents the n-th species that carries no plasmids, and $S_n^{\gamma_z}$ represents the n-th species that carries all plasmids at once.

Then we can define the following basic linear algebra operators:
Let $\overline{\gamma_j} = 1 - \gamma_j$ be the complement of γ_j.
Let $H(\gamma_i, \gamma_k) = \gamma_j \cdot \gamma_k = \sum_{\phi=1}^{p} \gamma_i[\phi]\gamma_k[\phi]$ be the dot product.

Several useful operations follow:
1. $H(\gamma_j, \gamma_j)$ is the identity operation, and will give the sum of all 1's in γ_j. Intuitively, this calculates the number of unique plasmids in a single population. For example, let $\gamma_j = [0\ 1\ 1]$:

$H(\gamma_j, \gamma_j) = [0 \quad 1 \quad 1] \cdot [0 \quad 1 \quad 1] = (0 \cdot 0) + (1 \cdot 1) + (1 \cdot 1) = 2.$

2. $H(\gamma_j, \gamma_k)$ is the number of elements shared between two vectors. Intuitively, this represents the number of unique plasmids carried between two populations. For example, let $\gamma_j = [0\ 1\ 0]$ and $\gamma_k = [0\ 1\ 1]$:
$H(\gamma_j, \gamma_k) = [0 \quad 1 \quad 0] \cdot [0 \quad 1 \quad 1] = 1.$

3. $H(\overline{\gamma_j}, \gamma_k)$ is the number of elements that are not in γ_j and in γ_k. Intuitively, this represents the number of plasmids that are in one population, but not the other. Thus, this operation calculates the number of potential conjugation interactions between two sets of plasmids γ_j and γ_k. For example:
$H(\overline{\gamma_j}, \gamma_k) = [1 \quad 0 \quad 1] \cdot [0 \quad 1 \quad 1] = 1$. A population carrying γ_k can therefore donate a single plasmid to a recipient carrying γ_j. Say instead, $\gamma_j = [1 \quad 0 \quad 0]$. Then $H(\overline{\gamma_j}, \gamma_k) = [0 \quad 1 \quad 1] \cdot [0 \quad 1 \quad 1] = 2$. This means a population carrying γ_k can donate one of two plasmids to the recipient carrying γ_j.

Using these rules, the differential equation for a unique population $\frac{dS_n^\gamma}{dt}$ can be generally represented in the following equation, and is broken down into five individual terms in the table below:

$$\frac{dS_n^\gamma}{dt} = \mu_n^\gamma S_n^\gamma \left(1 - \frac{\left(\sum_{i=1}^{N} \sum_{j=0}^{z} S_i^j\right)}{N_m}\right) + \eta_C \sum_{\forall k} S_n^{\gamma_k} \sum_{i=1}^{N} \sum_{\forall \rho} S_i^{\gamma_\rho} - S_n^\gamma \eta_C \sum_{i=1}^{N} \sum_{\forall q} S_i^{\gamma_q} H(\overline{\gamma}, \gamma_q) + \kappa \sum_{\forall \lambda} S_n^{\gamma_\lambda} - \kappa S_n^\gamma H(\gamma, \gamma) - D S_n^\gamma \quad \text{Eq 16}$$

See Supplementary Table 4 for a complete explanation of the equation terms.

Supplementary Table 1. Strains and plasmids used in this study

Name	Strain[1]	Plasmid	Description	Resistance[2]	Experiment temperature	References
B^0	DA26735 Eco lacIZYA::FRT, galK::mTagBFP2-amp[3]	F_{HR}	*1.* Chromosomal BFP<br>*2.* Chromosomal Amp^R<br>*3.* Helper F_{HR}	Amp^R, Tet^R	37°C	Strains from[3] and F_{HR} plasmid from[4].
B^K	DA26735 Eco lacIZYA::FRT, galK::mTagBFP2-amp[3]	1. F_{HR}<br>2. K (Kan^R, YFP, *oriT*)	*1.* See B^0<br>2. Mobilizable plasmid transferrable through F_{HR}	Amp^R, Tet^R , Kan^R	37°C	Strains from[3] and K and F_{HR} plasmid from[4].
B^{K-}	DA26735 Eco lacIZYA::FRT, galK::mTagBFP2-amp[3]	1. F_{HR}<br>2. K^- (Kan^R, YFP)	See B^0	Amp^R, Tet^R , Kan^R	37°C	Strains from[3] and K and F_{HR} plasmid from[4].
B^C	DA26735 Eco lacIZYA::FRT, galK::mTagBFP2-amp[3]	1. F_{HR}<br>2. C (Cm^R, mCherry, *oriT*)	*1.* See B^0<br>*2.* Mobilizable plasmid transferrable through F_{HR}	Amp^R, Tet^R , Cm^R	37°C	Strains from[3] and F_{HR} plasmid from[4]. Plasmid C from[5].
R^0	DA32838.Eco galK::cat-J23101-dTomato[3]	F_{HR}	*1.* Chromosomal dTomato<br>*2.* Chromosomal Amp^R<br>*3.* Helper F	Cm^R, Tet^R	37°C	Strains from[3] and K and F_{HR} plasmid from[4].
R^K	DA32838.Eco galK::cat-J23101-dTomato[3]	1. F_{HR}<br>2. K (Kan^R, YFP, *oriT*)	*1.* See R^0<br>*2.* Mobilizable plasmid transferrable through F_{HR}	Cm^R, Tet^R , Kan^R	37°C	Strains from[3] and K and F_{HR} plasmid from[4].
R^{K-}	DA32838.Eco galK::cat-J23101-dTomato[3]	1. F_{HR}<br>2. K^- (Kan^R, YFP)	See B^0	Cm^R, Tet^R , Kan^R	37°C	Strains from[3] and K and F_{HR} plasmid from[4].
Y^0	DA28100 Eco galK::cat-J23101-SYFP2[3]	F_{HR}	See B^0	Cm^R, Tet^R	30°C	Strains from[3] and K and F_{HR} plasmid from[4].
B^0 in multi species/plasmid experiment	DA28102 Eco galK::cat-J23101-mTagBFP2[3]	F_{HR}	See B^0	Cm^R, Tet^R	30°C	Strains from[3] and K and F_{HR} plasmid from[4].
ESBL donor	Three *E. coli* Isolates: Numbers 41, 168 and 193	ESBL plasmids	ESBL plasmids from clinical *E. coli* isolates ESBLs (Fig. 2D).	Amp^R, Ctx^R	30°C	ESBL isolates screened and characterized from[5].
R6K donor	*E. coli* strain k-12 J53, F^- met pro Azi^r	R6K	Strain containing the native RP4 self-transmissible plasmid.	Amp^R, Str^R	30°C	Obtained from from[5].

[1] SYFP2, mTagBFP2, and dTomato indicate yellow, blue, and red fluorescence, respectively
[2] Resistance abbreviations are defined as Tet^R (tetracycline), Cm^R (chloramphenicol), Kan^R (kanamycin), Amp^R (Ampcillin), Str^R (Streptomycin), and Ctx (cefotaxime)

RP4 donor	*E. coli* MC4100z1 (F^- *araD139 Δ(argF-lac)U169 rpsL150* (Str^R)*relA1 flbB5301 deoC1 ptsF25 rbs*R / RP4 IncPα Tra+*amp*^{R} *kan*^{R} *tet*^{R})	RP4	Strain containing the native RP4 self-transmissible plasmid.	Kan^R, Amp^R, Str^R	30°C	Obtained from from[5].
R388 donor	*E. coli* strain k-12 J53, F^- met pro Azi^r	R388	Strain containing the native RP4 self-transmissible plasmid.	Tm^R, Sm^R	30°C	Obtained from from[5].
PCU1 donor	*E. coli* DH5α*Pro"* (F^- *endA1 glnV44 thi-1 recA1 relA1 gyrA96 deo*R *nupG purB20* φ80d*lac*ZΔM15 Δ(*lacZYA-argF*)U169, hsdR17($r_K^- m_K^+$), $λ^-$)	PCU1	Strain containing the native PCU1 self-transmissible plasmid. Experiments performed at 37°C due to the high cost.	Amp^R	37°C	Provided by the Redinbo lab at UNC chapel hill.

Supplementary Table 2. Experimental estimates and modeling parameter values

Parameter and description	Experimental value	Experimental units	Modeling value	Modeling units	Reference
κ<br>Plasmid loss rate	5.2×10^{-4}	Hr^{-1}	0.001	Hr^{-1}	This paper (Supplementary Fig. 1A) and ref.[2]
α<br>Relative plasmid burden for S_1 (Supplementary Eq 3-4)	1. B^K modulated with Kan (0, 0.5, 2 μg/mL, Fig. 2B): [1.02, 0.97, 0.42]<br>2. B^C modulated with Cm (0,0.5, 2 μg/mL, Fig. 3B): [1.21, 0.89, 0.33]	Unitless	1. [1.02, 0.97, 0.42]<br>2. [1.13, 1.05, 0.45]	Unitless	This paper (Supplementary Fig. 1C)<br>This paper (Supplementary Fig. 4B)
α_1<br>Relative plasmid burden for S_1 (Supplementary Eq 7-10)	1. B^K modulated with Kan (0, 0.5, 2 μg/mL, Fig. 2B): [1.02, 0.97, 0.42]	Unitless	1. [1.02, 0.97, 0.42]	Unitless	This paper (Supplementary Fig. 4A)
α_2<br>Relative plasmid burden for S_2 (Supplementary Eq 7-10)	1. R^K modulated with Kan (0, 0.5, 2 μg/mL, Fig. 3A): [1.07 0.99 0.86]	Unitless	1. [1.03, 1.02, 0.9]	Unitless	This paper (Supplementary Fig. 4A)

α_K<br><br>Relative plasmid burden for S_1 (Supplementary Eq 11-14)	B^K modulated with Kan (0, 0.5, 2 μg/mL, Fig. 3C): [1.02, 0.97, 0.42]<br><br>B^K modulated with Cm (0, 0.5 2 μg/mL, Fig. 3C): [1.02, 1.035, 1.00]	Unitless	1. [1.02, 0.97, 0.42]<br><br>2. [1.02 1.02 1.02]	Unitless	This paper (Supplementary Fig. 4B)
α_C<br><br>Relative plasmid burden for S_1 (Supplementary Eq 11-14)	B^C modulated with Kan (0, 0.5, 2 μg/mL, Fig. 3C): [1.21, 1.5, 1.00]<br><br>B^C modulated with Cm (0, 0.5, 2 μg/mL, Fig. 3C): [1.21, 0.89, 0.33]	Unitless	1. [1.13, 1.13, 1.2]<br><br>2. [1.13 1.05 0.45]	Unitless	This paper (Supplementary Fig. 4B)
α_{CK}<br><br>Relative plasmid burden for S_{11} (Supplementary Eq 11-14)	B^{CK} modulated with Kan (0, 0.5, 2 μg/mL, Fig. 3C): [1.38 1.4 0.6]<br><br>B^{CK} modulated with Cm (0, 0.5, 2 μg/mL, Fig. 3C): [1.38 1.13 0.4]	Unitless	1. [1.3, 1.2, 0.42]<br><br>2. [1.3, 1.02, 0.35]	Unitless	This paper (Supplementary Fig. 4B)
μ_1<br><br>Growth rate of plasmid population for (B^K) S_1	B^K = 0.2-0.4 depending on temperature	Hr^{-1}	0.3	Hr^{-1}	This paper (Supplementary Fig. 3B)
μ_2<br><br>Growth rate of plasmid population for (R^K) S_2	R^K = 0.2-0.4 depending on temperature	Hr^{-1}	0.33	Hr^{-1}	This paper (Supplementary Fig. 3B)
1. α_B^{R6K}<br><br>2. α_B^{R388}<br><br>3. α_B^{RP4}<br><br>Relative plasmid burden for species B with each plasmid (Supplementary Eq 16)	1. 0.87<br><br>2. 0.84<br><br>3. 0.89<br><br>(Used in Fig. 3D-E, and Supplementary Fig. 5C)	Unitless	1. 0.87<br><br>2. 0.84<br><br>3. 0.89	Unitless	This paper (Supplementary Fig. 5B)

1. α_R^{R6K}<br>2. α_R^{R388}<br>3. α_R^{RP4}<br>Relative plasmid burden for species R with each plasmid (Supplementary Eq 16)	1. 0.75<br>2. 0.66<br>3. 0.72<br>(Used in Fig. 3D-E, and Supplementary Fig. 5C)	Unitless	1. 0.75<br>2. 0.66<br>3. 0.72	Unitless	This paper (Supplementary Fig. 5B)
1. α_Y^{R6K}<br>2. α_Y^{R388}<br>3. α_Y^{RP4}<br>Relative plasmid burden for species Y with each plasmid (Supplementary Eq 16)	1. 0.92<br>2. 0.86<br>3. 0.90<br>(Used in Fig 3D-E, and Supplementary Fig. 5C)	Unitless	1. 0.92<br>2. 0.86<br>3. 0.90	Unitless	This paper (Supplementary Fig. 5B)
1. μ_{s1}<br>2. μ_{s2}<br>3. μ_{s3}<br>Growth rate of plasmid-free species in multi-plasmid multi-species experiment (R^0,B^0,Y^0, Supplementary Eq 16)	1. 0.217<br>2. 0.219<br>3. 0.18	Hr^{--1}	1. 0.217<br>2. 0.219<br>3. 0.18		This paper (Supplementary Fig. 5B)
N_m<br>Carrying capacity	~1x10^9	Cells/mL	1	Unitless	This paper
D<br>Dilution rate	0.05	Hr^{-1}	0.05	Hr^{-1}	This paper
η and η_C	η = 2.5x10^{-11}	$Cells^{-1}hr^{-1}$	η_C = 2.5x10^{-2}	Hr^{-1}	This paper

Conjugation efficiency					(Supplementary Fig. 3A) and ref [5]
S_i Initial starting density	~80	Cells/mL	1x10^{-6}	Unitless	This paper

**Plasmid**	**Origin**	**Cost**	$\boldsymbol{\eta_{Crit}}$	**Efficiency (No inhibition)** (cells^{-1}hr^{-1})	**Predicted persistence (no inhibition)**	**Fold-decrease of efficiency with inhibition**	**Predicted persistence (with inhibition)**	**Criteria met (No inhibition)**
K	*oriT* mobile F	1.02	2.4x10^{-3}	2.5x10^{-15}	Y	2.96	N	Y

β	10^{-5}	Hr^{-1}	10^{-5}	Hr^{-1}	Refs[6,1]

Supplementary Table 3: Plasmid parameter summary and predictions

C	*oriT* mobile F	1.21	9.1x10^{-3}	1.09x10^{-14}	Y	1.54	Y	Y
41[7]	incN/incF	1.36	0.02	1.80x10^{-14}	Y	12.43	N	Y
168[7]	incF/incN	0.95	-2x10^{-4}	5.94x10^{-15}	Y	53.42	N	Y
193[7]	incI/incF	0.90	-4x10^{-3}	2.38x10^{-15}	Y	3.31	Y	Y
RP4	incP	0.81	-4x10^{-3}	1.76x10^{-12}	Y	13.06	Y	Y
R6K	incX	1.03	-0.01	9.19x10^{-14}	Y	2.46	Y	Y
PCU1	incN	1.21	0.94	1.45x10^{-13}	Y	13.09	N	Y
R388	incW	0.66	-0.16	1.94x10^{-12}	Y	2.49	Y	Y

Supplementary Table 4. Generalized conjugation model terms

Term	Representation	Additional definitions and description
Logistic growth	$\mu_n^{\gamma} S_n^{\gamma} \left(1 - \frac{\left(\sum_{i=1}^{N} \sum_{j=0}^{z} S_i^j\right)}{N_m}\right)$	There are a total of $N2^p$ populations
Contribution from conjugation	$\eta_C \sum_{\forall k} S_n^{\gamma_k} \sum_{i=1}^{N} \sum_{\forall \rho} S_i^{\gamma_\rho}$	**Recipients:** Define k to be the indices corresponding to a subset of vectors in $\{\gamma_j\}$ $(\{\gamma_k\} \subseteq \{\gamma_j\})$ such that each vector has one unique plasmid difference to γ, and has less total plasmids (number of

		plasmids carried by $S_n^{\gamma_k}$ is less than the number of plasmids carried by S_n^{γ} for all k).<br><br>**Donor:** Define ρ to be the indices corresponding to a subset of vectors in$\{\gamma_j\}$ consisting of every vector combination that carries the missing plasmid as defined by γ_k.
Loss from conjugation	$-S_n^{\gamma}\eta_C \sum_{i=1}^{N} \sum_{\forall q} S_i^{\gamma_q} H(\bar{\gamma}, \gamma_q)$	**Recipients:** The population S_n^{γ} will gain a plasmid through conjugation<br><br>**Donor:** Define *q* to be the indices corresponding to a subset of vectors in$\{\gamma_j\}$) that has at least one unique plasmid not contained by S_n^{γ}.
Contribution from plasmid segregation	$\kappa \sum_{\forall \lambda} S_n^{\gamma_\lambda}$	Define λ to be the indices corresponding to a subset of vectors in$\{\gamma_j\}$ consisting of every vector combination that has every single plasmid in γ plus exactly one more plasmid $(\{\gamma + 1\})$.
Loss from plasmid segregation	$-\kappa S_n^{\gamma} H(\gamma, \gamma)$	S_n^{γ} loses each of the plasmids in γ with the rate constant κ
Dilution	$-DS_n^{\gamma}$	First order kinetics for dilution

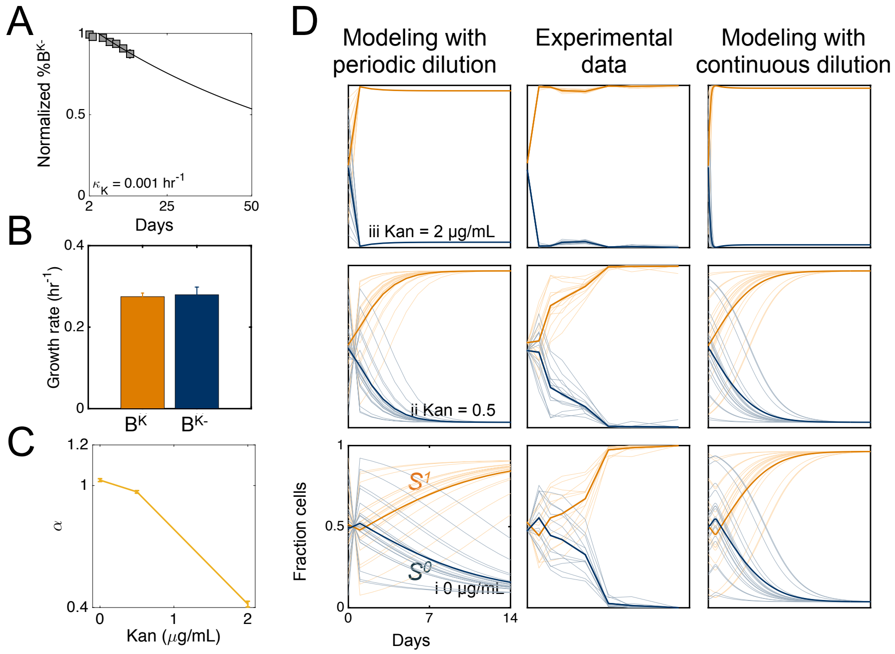

Supplementary Figure 1: Experimentally estimating the plasmid parameters

A. **Estimating plasmid loss rate using B^{K-}.** 100% B^{K-} culture was propagated daily in the presence of 50 μg/mL Kan or nothing, using the same protocol as the main experiments (Fig. 2B-C). Flow cytometry measurements obtained every day to quantify the percentage of B^{K-} cells as a fraction of all BFP expressing cells. Y-axis is the fraction of B^{K-} normalized by B^{K-} with Kan. Black line indicates exponential decay fit $y = x_1 e^{-x_2 t}$, where the calculated plasmid loss rate constant for K is $\kappa_K = \frac{x_2}{24}$ hr^{-1} and is printed in the bottom left of the graph. Eight well replicates were used for flow measurements, and repeated at least twice for reproducibility

B. **Negligible effect of *oriT* on growth rate.** Growth rates of B^K and B^{K-} were obtained by diluting cells 10,000X from overnight culture in M9 media (see **Methods**), and grown at 37°C for at least 14 hours. Growth curves were log-transformed and the linear portion was fit to a regression. Measurements were performed in quadruplicates, and error bars are standard deviation. *oriT* did not significantly influence the growth rate of B^K (P>0.5, two-sided t-test).

C. **Estimating plasmid burden α at various antibiotic concentrations.** Growth curves of B^0 and B^K were obtained as described in (B). α was obtained by normalizing the rates obtained from the species without the plasmid by the growth rate of the corresponding species with the plasmid (e.g. $\frac{B^0}{B^K}$). Technical replicates performed in quadruplicate, and repeated at least three times. Error bars represent standard deviation.

D. **Conclusions hold regardless of modeling dilution continuously or periodically**. To determine whether periodic dilutions were critical to recapitulate experimental results, we modeled the data using both continuous D (right column, 0.05 hr^{-1}) and discrete transfer events (left column, once every 24 hours), and compared it to the experimental data (middle column). Periodic or continuous dilutions appear qualitatively identical. The shaded lines are 20 simulation replicates with varied initial cell densities randomly chosen from a normal distribution. The dark blue line is the average of all time courses for plasmid-free cells (S^0), and the dark orange line is the average of all plasmid-carrying cells (S^1). i-iii is decreasing α from costly to beneficial, α = 1.02, 0.97, 0.42 respectively.

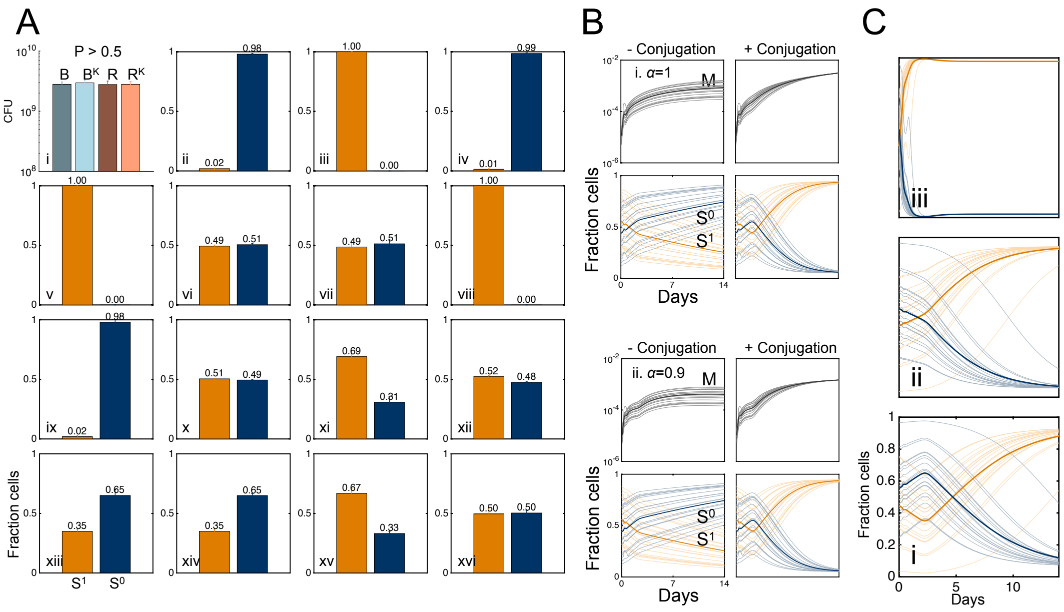

Supplementary Figure 2: Calibration for one-species one-plasmid system

A. Flow cytometer calibration for red, yellow, and blue fluorescence. (i) CFU quantified for R^0, B^0, R^{K-}, and B^{K-} show statistically indistinguishable densities (P >0.5, two-sided t-test). Volume ratios were therefore used for flow calibration. (ii-xvi) Overnight cultures were diluted into autoclaved deionized water at indicated ratios for dilution factors of high (400X) and low (1,000X) values. Gating based on 10 μL sample volume was chosen such that measurements were accurate within 5% of the predicted fluorescence for each mixture. Three technical replicates for each dilution were obtained. Error bars are standard deviation from all conditions. Orange and blue represents the sum of all plasmid-bearing and plasmid-free cells, respectively. The measured percentages are listed above each bar, and expected ratios were as follows for R^0 B^0 R^K and B^K respectively (ii : xvi): (1,0,0,0), (0,0,1,0), (0,1,0,0), (0,0,0,1), (.5,0,.5,0), (0,.5,0,.5), (.5,.5,0,0), (0,0,.5,.5), (.5,0,0,.5), (0,.5,.5,0), (.33,.33,.33,0), (.33,.0,.33,.33), (0,.33,.33,.33), (.33,0,.33,.33), (.33,.33,0,.33), and (.25,.25,.25,.25)

B. Effects of compensatory mutations will negligibly influence the overall dynamics. A third population (mutant, *M*) transitions from S^1 at a rate β. *M* is assumed to either have neutral plasmid burden ($\alpha = 1$, i), or beneficial ($\alpha = 0.9$, ii). Dynamics were analyzed with and without conjugation (right and left columns, respectively). Regardless of α, even with a high mutation rate (10^{-5} hr^{-1})[6], the plasmid cannot persist without conjugation. With conjugation, mutants are present at low frequencies. Solid blue, orange, and gray lines represent the average fraction of S^0, S^1, and *M* respectively, and shaded lines represent randomized initial conditions (see Fig. 2B).

C. Removing noise in conjugation efficiency does not change overall results. The dark blue line is the average of all time courses for plasmid-free cells (S^0), and the dark orange line is the average of all plasmid-carrying cells (S^1). i-iii is decreasing α from costly to beneficial, α = 1.02, 0.97, 0.42 respectively. For (B) and (C), x-axis is time in days, the y-axis is fraction of cells, and the shaded lines are 20 simulation replicates with varied initial cell densities randomly chosen from a normal distribution.

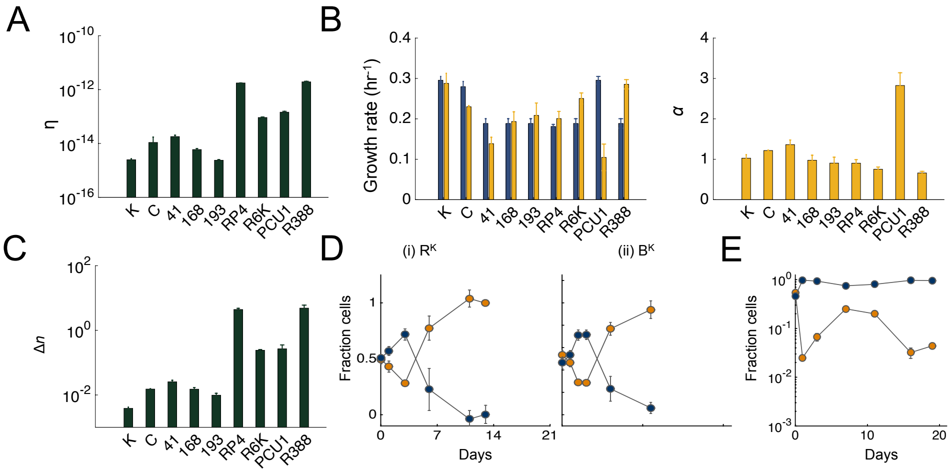

Supplementary Figure 3: Estimating parameters for native plasmids.

A. Estimating conjugation efficiency for all native plasmids. R^0 was used as recipient for all measurements, except for C where B^0 was used instead. Native strains for all ESBL plasmids were used as the donors, and strains with compatible resistance to both the plasmid being tested, and R^0 were used as donors for the remaining plasmids (Supplementary Table 1 for details). Equal fractions of donor and recipient were mixed for one hour at room temperature. Cells were then diluted between 40-10^4X, depending on the plasmid, and spread on double selection plates to quantify the transconjugants (Carb+Cm for all plasmids except for R388 (Cm+Tm), RP4 and K (Cm+Kan)). Y-axis is $\eta = \frac{T}{DR\Delta t}$, where T, D, and R are the densities of the transconjugant, the donor, and the recipient, respectively.

B. Growth rates of plasmid-bearing (yellow bars) and plasmid-free (blue bars) cells. Overnight cultures were diluted 10,000X and measured at either 37°C (K, C, and PCU1) or 30°C (RP4, R388, R6K, #41, #168, and #193), consistent with the long-term conditions. Growth curves are log-transformed and fit to a linear regression. Left panel is growth rate estimates, and right panel shows α (e.g. normalized to the growth rate without plasmid).

C. Estimated critical conjugation efficiency η_{Crit} suggests persistence for all 9 plasmids. $\Delta n = \eta_C - \eta_{Crit} > 0$ indicates persistence. η_{Crit} estimates are based on direct measurements of κ=0.001 hr^{-1} (Supplementary Table 1A), α (Supplementary Table 3B), and compared to η_C (Supplementary Table 3A normalized by N_m=1x10^9). D is assumed to be 0.05 hr^{-1} for all estimates.

D. R and B exhibit qualitatively similar long-term plasmid dynamics. Dynamics of plasmid K was performed with both B (B^0/B^K) and R (R^0/R^K) following the same protocol as all other long-term experiments (see **Methods** section, 'Conjugation dynamics for native plasmids'). Overall dynamics are qualitatively the same between the two strains.

E. PCU1 persisted but exhibited weaker coexistence. Dynamics of plasmid PCU1 (same as data from Fig. 2D viii) is plotted on a log-scale to demonstrate the persistence over 19 days, despite weaker degree of coexistence (and high cost).

All measurements use background species R, except for plasmid C, which uses B instead due to resistance incompatibilities with R (both Cm^R). Measurements were performed in at least four replicates, and error bars represent the standard deviation of the growth rates averaged across all replicates (except D, where replicates were performed at least twice).

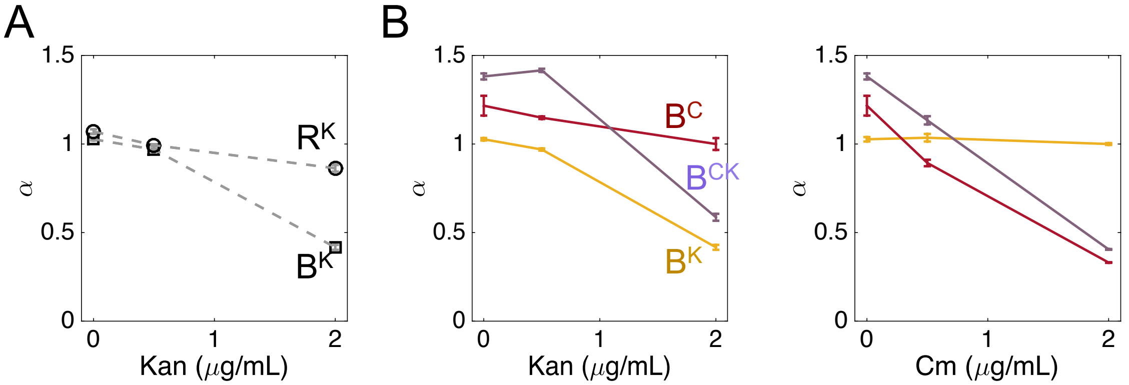

Supplementary Figure 4: Parameter estimation for multiple populations and plasmids

A. ***α* estimates for the two-species one-plasmid experiment** (Fig. 3A). Overnight cultures (16 hours, LB broth, 37°C) were diluted 10,000X and grown in the presence of 0, 0.5, or 0.2 μg/mL Kan. Circles represent R population and squares represent B population. α is estimated according to **Methods** section, 'Quantifying modeling parameters α η and κ'; listed in Supplementary Table 2.

B. ***α* estimates for the one-species two-plasmid experiment** (Fig. 3C). B, B^K, B^C, or B^{CK} are grown in the presence of 0, 0.5, or 0.2 μg/mL Kan (left) or Cm (right), as described in (A). α is estimated according to **Methods** section (see 'Quantifying modeling parameters α η and κ'); and listed in Supplementary Table 2.

For both A and B, Measurements were performed in at least four replicates, and error bars represent the standard deviation of the growth rates averaged across all well replicates.

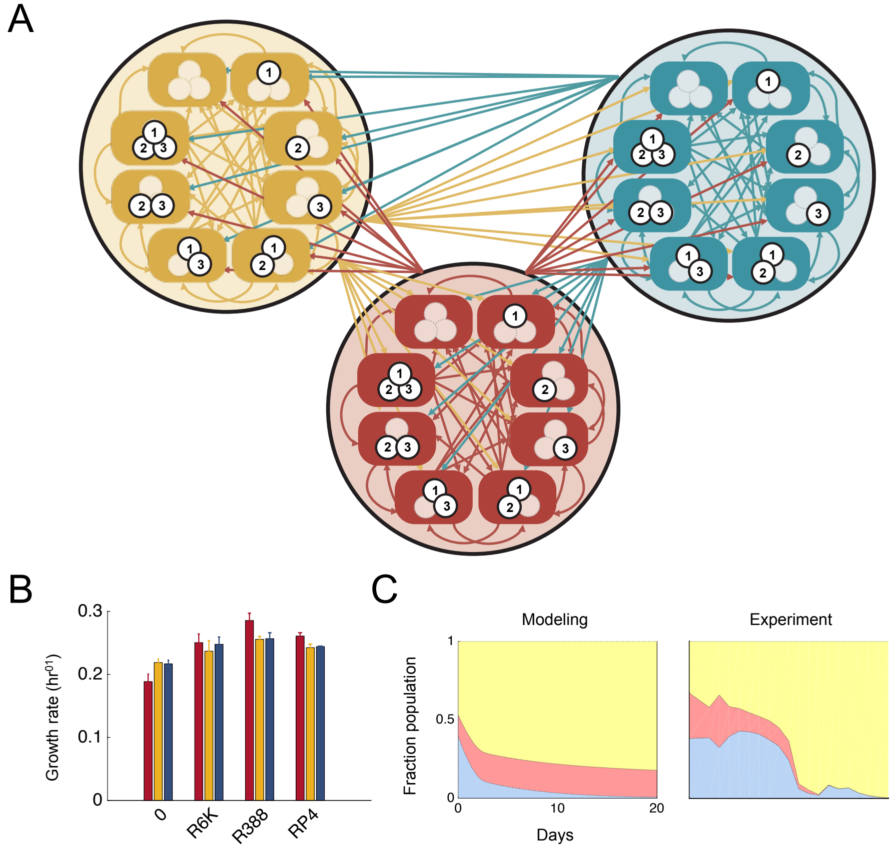

Supplementary Figure 5: Quantifying parameters for inhibition strategies

A. Diagrammatic representation of three-population three-plasmid community. The model consists of 24 populations, where each species (defined by unique η_{Crit}) consists of 8 sub-populations consisting of each unique combination of plasmids. Each species is represented by the color red, blue and yellow respectively, and the lines connect each population to their potential donor or recipient. Plasmid loss is omitted for simplicity.

B. Growth rates for populations used in the multi-species multi-plasmid experiment (Fig. 3E, R^0 B^0 Y^0 R^{R6K} B^{RP4} Y^{R388}). To obtain a baseline assessment of cost, growth rates are estimated for each species (R, B, and Y) carrying each plasmid (R6K, RP4, and R388). Bar color indicates the corresponding species (e.g. red, blue, and yellow for R^0, B^0, and Y^0 respectively). Grouped bars indicate different plasmid (labeled), where 0 indicates no plasmid. Growth rates are estimated according to **Methods** section (see 'Quantifying modeling parameters α η and κ'); α values are listed in Supplementary Table 2.

C. Overall population dynamics of R^0 B^0 and Y^0 in the absence of any plasmid. The shaded color (red for R^0, blue for B^0, and yellow for Y^0). **Modeling** (left): x-axis is time in days and y-axis is the fraction of the three species (S_1 S_2, S_3) without any plasmid. The shaded color indicates the total fraction of each species. **Experiment** (right): R^0, Y^0, and B^0 are mixed in equal fraction and propagated daily with a dilution of 10,000X. Y^0 outcompetes the other populations, which is consistent with growth rate measurements (Supplementary Fig. 5B, see **Methods** section 'Conjugation dynamics for native plasmids').

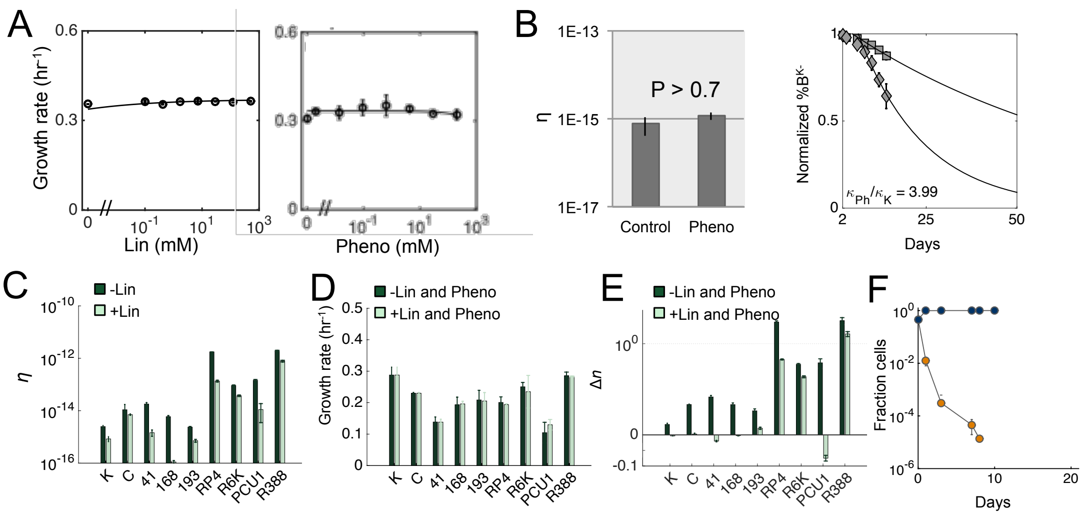

Supplementary Figure 6: Quantifying parameters for inhibition strategies

- **A.** *Left*: **Linoleic acid (Lin) did not inhibit growth of B^0 strain**. Concentrations of Lin used were 0, 0.01, 0.068, 0.46, 3.16, 21.54, 146.78, 1000 mM. *Right*: Pheno did not inhibit growth of B^0. Concentrations of phenothiazine tested were 0, 0.002, 0.14, 0.093, 0.63, 4.31, 29.36, 200.0 μM. See **Methods** for growth rate estimation.
- **B.** **Measuring effects of Pheno on conjugation efficiency and plasmid loss**. *Left:* R^0 was used as recipient and B^C as donor. 120 μM of Pheno did not affect the conjugation efficiency (P > 0.7, two-sided t-test). *Right:* B^{K-} was propagated daily with 50 μg/mL Kan, nothing, or 120 μM Pheno using the same protocol as previously described (see **Methods**). Y-axis is the fraction of B^{K-} normalized by the Kan control. Black line indicates exponential decay fits $y = x_1 e^{-x_2 t}$. Pheno increases the rate loss constant by ~4-fold (κ_{P_h}).
- **C.** **Measuring conjugation efficiency in the presence of Lin.** Same donor and recipient setup, and experimental conditions, were used as in Supplementary Fig. 3A. 3.25 mM Lin used in overnight culture and during conjugation exposure. Cells were spread to a net-dilution fold of anywhere from 40-10^4X onto double selection plates, and CFU counted the following day.
- **D.** **Growth rates of each population in the presence of Lin and Pheno (light green bars) were not significantly different from the control (dark green bars)** (P>0.2, two-sided t-test). Growth rate estimates are performed the same as previously described.
- **E.** **Estimated critical conjugation efficiency η_{Crit} in the presence of Lin and Pheno.** Lin and Pheno were predicted to reverse 4/9 plasmids tested ($\Delta n < 0$), and varying degrees of suppression for the rest. Dark green indicates without intervention, and light green indicates updated parameter estimates in the presence of both Lin and Pheno. Error bars represent standard deviations from at least four replicates, and propagated when necessary (η_{Crit}).
- **F.** **Plasmid elimination of PCU1.** Compared to Fig. 2D viii, plasmid PCU1 is eliminated < 10 cells/mL when treated with Lin and Pheno (See **Methods**).

Error bar represents standard deviation from four-six replicates for all CFU and growth rate experiments.

Supplementary References

1 De Gelder, L. *et al.* Combining mathematical models and statistical methods to understand and predict the dynamics of antibiotic-sensitive mutants in a population of resistant bacteria during experimental evolution. *Genetics* **168**, 1131-1144, doi:10.1534/genetics.104.033431 (2004).

2 Gullberg, E. *et al.* Selection of Resistant Bacteria at Very Low Antibiotic Concentrations. *PLoS Pathog* **7**, doi:10.1371/journal.ppat.1002158 (2011).

3 Dimitriu, T. *et al.* Genetic information transfer promotes cooperation in bacteria. *Proceedings of the National Academy of Sciences*, 201406840, doi:10.1073/pnas.1406840111 (2014).

4 Lopatkin, A. J. *et al.* Antibiotics as a selective driver for conjugation dynamics. *Nature Microbiology*, 16044, doi:10.1038/nmicrobiol.2016.44 (2016).

5 Stewart, F. M. & Levin, B. R. The population biology of bacterial plasmids: a priori conditions for the existence of conjugationally transmitted factors. *Genetics* **87**, 209-228 (1977).

6 Schulz zur Wiesch, P., Engelstädter, J. & Bonhoeffer, S. Compensation of Fitness Costs and Reversibility of Antibiotic Resistance Mutations. *Antimicrobial Agents and Chemotherapy* **54**, 2085-2095, doi:10.1128/aac.01460-09 (2010).

7 Kanamori, H. *et al.* Whole-Genome Sequencing of Multidrug-Resistant Escherichia coli Isolates From Community Hospitals in North Carolina, 2010–2015. *Open Forum Infectious Diseases* **3**, 200-200, doi:10.1093/ofid/ofw172.67 (2016).
